# Daily laxative therapy reduces organ dysfunction in mechanically ventilated patients: a phase II randomized controlled trial

**DOI:** 10.1186/s13054-015-1047-x

**Published:** 2015-09-16

**Authors:** Rodrigo Palacio de Azevedo, Flávio Geraldo Resende Freitas, Elaine Maria Ferreira, Luciano Cesar Pontes de Azevedo, Flávia Ribeiro Machado

**Affiliations:** Disciplina de Anestesiologia, Dor e Terapia Intensiva, Universidade Federal de São Paulo, Rua Napoleão de Barros, 715 - 6° andar - Vila Clementino, CEP: 04024-002 São Paulo, SP Brazil

## Abstract

**Introduction:**

Constipation is a common problem in intensive care units. We assessed the efficacy and safety of laxative therapy aiming to promote daily defecation in reducing organ dysfunction in mechanically ventilated patients.

**Methods:**

We conducted a prospective, randomized, controlled, nonblinded phase II clinical trial at two general intensive care units. Patients expected to remain ventilated for over 3 days were randomly assigned to daily defecation or control groups. The intervention group received lactulose and enemas to produce 1–2 defecations per day. In the control group, absence of defecation was tolerated up to 5 days. Primary outcome was the change in Sequential Organ Failure Assessment (SOFA) score between the date of enrollment and intensive care unit discharge, death or day 14.

**Results:**

We included 88 patients. Patients in the treatment group had a higher number of defecations per day (1.3 ± 0.42 versus 0.7 ± 0.56, *p* < 0.0001) and lower percentage of days without defecation (33.1 ± 15.7 % versus 62.3 ±24.5 %, *p* < 0.0001). Patients in the intervention group had a greater reduction in SOFA score (–4.0 (–6.0 to 0) versus –1.0 (–4.0 to 1.0), *p* = 0.036) with no difference in mortality rates or in survival time. Adverse events were more frequent in the treatment group (4.5 (3.0–8.0) versus 3.0 (1.0–5.7), *p* = 0.016), including more days with diarrhea (2.0 (1.0–4.0) versus 1.0 (0–2.0) days, *p* < 0.0001). Serious adverse events were rare and did not significantly differ between groups.

**Conclusions:**

Laxative therapy improved daily defecation in ventilated patients and was associated with a greater reduction in SOFA score.

**Trial registration:**

Clinical Trials.gov NCT01607060, registered 24 May 2012.

**Electronic supplementary material:**

The online version of this article (doi:10.1186/s13054-015-1047-x) contains supplementary material, which is available to authorized users.

## Introduction

Constipation is a frequent problem in intensive care units (ICUs) and has often been overlooked [1]. A number of factors can contribute to constipation in critically ill patients, including immobility, dehydration and the use of sedatives, opioids and vasopressors [1–3]. The reported incidence of constipation in this population varies from 5 to 84 % [4–9].

Constipation may be part of a broader context of acute intestinal dysfunction [10]. Recent studies have identified constipation as an independent prognostic factor in critically ill patients [1, 2, 9, 11, 12], suggesting that its treatment would improve outcome [13]. Bowel dysfunction can lead to complications, such as bacterial translocation, abdominal distension, delayed gastric emptying, vomiting, intestinal obstruction and perforation [14–17] and may be associated with worsened prognosis [18]. However, it remains unclear whether constipation is merely a marker of disease severity or an organ dysfunction to be treated.

Given the absence of relevant studies, we conducted this phase II study to assess the effects of a laxative therapy protocol aimed at promoting daily defecation on organ dysfunction in mechanically ventilated patients. We hypothesized that daily defecation would reduce bacterial translocation and improve intra-abdominal pressure by reducing abdominal distention and enhancing gastric emptying, thus contributing to a reduction in overall organ dysfunction. We also aimed to evaluate the efficacy of the protocol in promoting daily defecation and the safety of this intervention by assessing its associated adverse events.

## Methods

### Study design

The study was an investigator-initiated, prospective, randomized, controlled, nonblinded clinical trial of critically ill, mechanically ventilated patients admitted to two general, mixed ICUs (9 and 17 beds) at a Brazilian university hospital. We randomly assigned patients in a 1:1 ratio to a daily defecation group or to a control group in blocks of 10 per unit using sealed opaque envelopes. The study team was responsible for generating the envelopes, enrolling participants and assigning them to the groups. As the failure to produce daily defecation in the intervention group would result in the use of enemas, it was not possible to blind the intervention. Thus, all members of the study team and the assistant physicians were aware of group assignment. The study was registered in Clinical Trials (NCT01607060). The Ethical Committee of the Federal University of Sao Paulo approved the study (number 0844/08), and all patients or their legal representative gave their written informed consent.

### Patients

We enrolled mechanically ventilated patients over 18 years of age during the first 72 hours after ICU admission who were expected to remain in the ICU and on mechanical ventilation (MV) for more than 3 days and who were receiving at least 20 % of their target calories as enteral nutrition. We excluded those patients with unstable fractures that may have resulted in limitation of bed mobility, those who were pregnant, those with gastrointestinal ostomy (colostomy, ileostomy or jejunostomy) or any gastrointestinal disease associated with diarrhea, those with chronic liver disease, neuromuscular diseases, spinal cord injury, severe hemodynamic instability, survival expectancies of less than 24 hours, those under palliative care and those previously included in the study.

### Study protocol

All patients received treatment until day 28 after inclusion, ICU discharge or death, whichever came first. The protocol is described in detail in Additional file 1. Briefly, the intervention group received lactulose (667 mg/ml; Lactulona®, Daiichi Sankyo, Barueri, SP, Brazil) at an initial dose of 20 ml every 8 hours. The goal was for the patient to produce 1–2 defecations per day. Lactulose was discontinued in patients with diarrhea. In the control group, the absence of defecation was tolerated for up to 5 days, unless symptoms of obstipation were present. Rectal examination and enemas were prescribed as needed, and lactulose use was discouraged. The general treatments and procedures for weaning from MV for both groups were based on local protocols. Nutritional support was also based on local protocols and is briefly described in Additional file 1.

### Measurements

Our primary outcome was the change in Sequential Organ Failure Assessment (SOFA) score between the date of enrollment (day 0) and the day of ICU discharge, death or day 15 of the study, whichever came first (termed ΔSOFA). The secondary outcomes were the ventilator-free days within 28 days, and the length of ICU and hospital stays. We determined the 28-day, ICU and hospital mortality rates. For those patients discharged from the hospital before day 28, survival status was determined by consulting with the hospital register for reports of outpatient clinic visits, hospital readmissions or phone calls. We used the daily Therapeutic Intervention System Score (TISS)-28 [19] to assess nurse workload, and the data were reported as the mean values of the daily TISS-28 and the total TISS-28 during the study period. We also recorded the occurrence of new infections, considering only pneumonia, catheter-related bloodstream infections and urinary tract infections, the presence of bacteremia, new episodes of severe sepsis or of septic shock, and the occurrence of new organ dysfunctions, according to the definitions available in Additional file 1.

For all patients, we recorded the general descriptive data as described in Additional file 1. The effectiveness of the protocol was assessed throughout the treatment period, primarily by the number of defecations per day, which was defined as the total number of defecations occurring during the treatment period divided by the total days of observation. We measured the percentage of days without evacuation during the observation period and the time to the first defecation. For patients who did not defecate during the treatment period, we considered the number of hours of observation. The percentage of days in which enemas were used and the mean daily dose of lactulose were also recorded. We also assessed the frequency of constipation, defined as the absence of stools for more than 3 consecutive days without mechanical obstruction according to the unit’s protocol for obstipation management.

We assessed adverse events (AEs) during the entire study period. We considered only those events with higher potential associations with the laxative therapy. Thus, we focused on the occurrences of diarrhea, vomiting, abdominal distension, dermatitis, pressure ulcers, electrolyte disturbances (hypernatremia, hypokalemia and hypomagnesemia), gastric reflux, gastrointestinal bleeding and Ogilvie's syndrome (see Additional file 1 for details). We considered diarrhea as the presence of three or more defecations in a given day as previously defined [20]. Serious adverse events included Ogilvie's syndrome, any gastrointestinal bleeding, hypernatremia ≥160 mEq/l, hypokalemia ≤2.5 mEq/l or hypomagnesemia ≤1.0 mEq/l and any other serious event that may have been related to the protocol in the opinion of the attending physician.

### Statistical analysis

The sample size calculation was based on a predicted difference of 2.5 points in the ΔSOFA score considering a power of 80 % with an error of 0.05. Thirty-six patients per group would have been necessary. However, we adjusted the sample by 20 % expecting a non-normal distribution of this variable. Thus, the final sample size was 44 patients per group, totaling 88 patients.

All variables were subjected to the Kolmogorov-Smirnov test to assess their distributions. We described the categorical variable as number and percentage. The continuous variables were described as medians and their respective 25th and 75th percentiles or as the mean ± standard deviation, according to their distributions. To assess the primary and secondary outcomes we constructed a generalized linear model with gaussian family with identity link, quasipoisson with log link or binomial with log link as appropriate. All models were controlled by Acute Physiologic Chronic Health Evaluation (APACHE) score and SOFA score at baseline. The results were expressed by relative risk or mean difference between groups, with their respective 95 % confidence intervals, estimated with the delta method from a quasipoisson regression considering median APACHE and SOFA scores at baseline.

The intention-to-treat analysis included all patients randomly assigned to the intervention group or to the control group. The per-protocol analysis included only those patients who remained under MV for at least 3 days. We used the SPSS 19.0 package for Windows (IBM, Chicago, Illinois, USA) and R 3.1.1 (R Core Team, 2014). All statistical tests were two-tailed, and the statistical significance was set at 0.05.

## Results

**Fig. 1:**
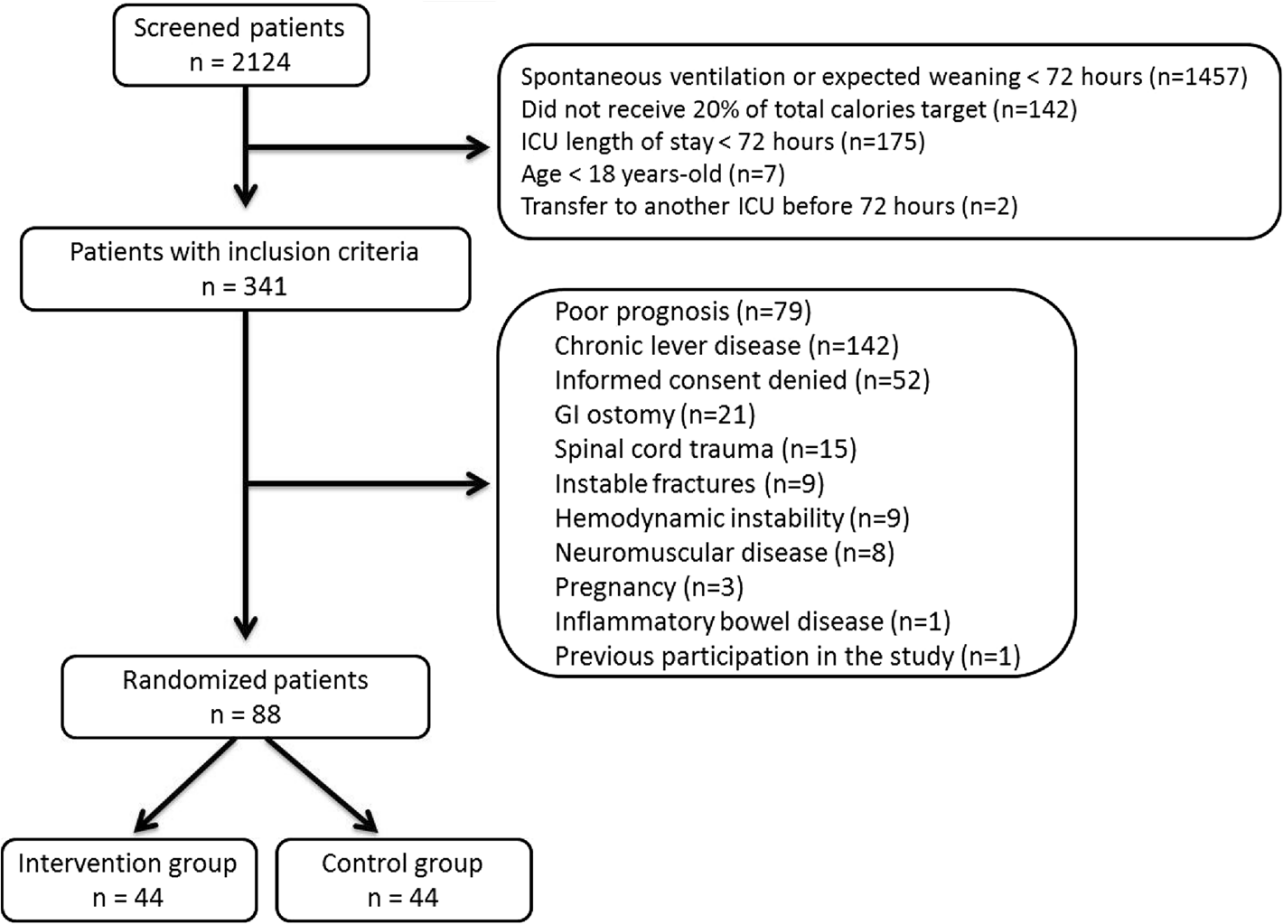
Study flow chart. *GI* gastrointestinal, *ICU* intensive care unit

From September 2008 to May 2012, 2124 patients were admitted to the study ICUs. Of these patients, 350 met the inclusion criteria and 88 patients were randomized. Figure 1 demonstrates the flowchart of the study. One patient from the intervention group denied to continue the participation 20 days after inclusion and at 2 days before ICU discharge but allowed for the use of the data. Table 1 shows the baseline data showing no significant differences between the groups.

**Table 1:** Baseline characteristics of the patients in both study groups

Variable	All patients	Intervention	Control	*p* value
	(n = 88)	(n = 44)	(n = 44)	
Male gender	54 (61.4)	29 (65.9)	25 (56.8)	0.381
Age, years	51.2 ± 19.6	50.4 ± 18.5	52.0 ± 20.8	0.697
Admission category				0.373
Medical	30 (34.1)	13 (29.5)	17 (38.6)	
Elective surgery	1 (1.1)	0	1 (2.3)	
Emergency surgery	57 (64.8)	31 (70.5)	26 (59.1)	
ICU cause of admission				0.822
Polytrauma	21 (23.9)	12 (27.3)	9 (20.5)	
Sepsis	20 (22.7)	10 (22.7)	10 (22.7)	
Intracranial hemorrhage	14 (15.9)	7 (15.9)	7 (15.9)	
TBI	11 (12.5)	6 (13.6)	5 (11.4)	
Respiratory failure	5 (5.7)	3 (6.8)	2 (4.5)	
Other	17 (19.3)	6 (13.6)	11 (25.0)	
Sepsis at enrollment	47 (53.4)	22 (50.0)	25 (56.7)	
Severe sepsis	14 (15.9)	4 (9.1)	10 (22.7)	0.103
Septic shock	33 (37.5)	18 (40.9)	15 (34.0)	
Comorbidities				
Hypertension	32 (36.4)	16 (36.4)	16 (36.4)	1.000
Diabetes mellitus	16 (18.2)	7 (15.9)	9 (20.5)	0.580
Stroke	8 (9.1)	4 (9.1)	4 (9.1)	1.000
Cancer	7 (8.0)	4 (9.1)	3 (6.8)	0.694
COPD	4 (4.5)	1 (2.3)	3 (6.8)	0.306
APACHE II, points	19.5 (15.0–22.0)	20.0 (16.0–24.0)	17.5 (14.0–22.0)	0.075
SOFA at enrollment, points	7.5 (6; 10)	9 (6–11)	7 (6–9)	0.294
TISS-28 at enrollment	37.5 (32.2–41.7)	36.0 (33.0–40.5)	38.0 (31.0–42.0)	0.738
Lactate at enrollment, mmol/l	1.7 (1.3–2.1)	1.6 (1.2–2.1)	1.7 (1.3–2.1)	0.512
Time to ICU admission, days	1.0 (0.7–3.0)	1.0 (0–3.0)	2.0 (1.0–4.0)	0.388
Time to enrollment, hours	50.5 (32.0–66.0)	46.0 (33.0–66.0)	51.0 (30.0–67.0)	0.897

*p* values determined by Chi-square test, Student’s *t*-test and Mann-Whitney test as appropriate

Time to ICU admission = time between hospital admission and ICU admission

Time to enrollment = time between ICU admission and study enrollment

Results are expressed as number (%), mean ± standard deviation, or median (25th–75th percentile)

*APACHE II* Acute Physiology and Chronic Health Evaluation II, *COPD* chronic obstructive pulmonary disease, *ICU* intensive care unit, *SOFA* Sequential Organ Failure Assessment, *TBI* traumatic brain injury, *TISS-28* Therapeutic Intervention Scoring System-28

The data pertaining to the effectiveness of the protocol are presented in Table 2. Treatment with lactulose was effective in promoting daily defecation. The patients included in the treatment group had a higher defecation rate, a lower time to first defecation, a lower percentage of days without defecation as well as a lower frequency of constipation throughout the study. Only four patients in the intervention group (9.1 %) did not have defecation for 4 consecutive days and three of them were not concurrently receiving enteral nutrition because of surgical interventions. For the intervention group, the mean duration of lactulose use was 15.2 ± 7.95 days, and the mean daily dose of lactulose was 43.5 ± 20.55 ml. Only one patient in the control group used a single dose of lactulose during the entire treatment period.

**Table 2:** Effectiveness of the protocol

Variable	Intervention	Control	*p* value
	(n = 44)	(n = 44)	
Number of defecation per day^a^	1.3 ± 0.42	0.7 ± 0.56	<0.001
Time to the first defecation, hours^b^	14.5 (4.5–24.0)	96.0 (50.5–127.5)	<0.001
Days without defecation, % days	33.1 ± 15.7	62.3 ± 24.5	<0.001
Constipation, number of patients^c^	4 (9.1 %)	32 (72.7 %)	<0.001
Enema administration, % days	21.1 (10.8–28.4)	4.5 (0–11.6)	<0.001

*p* values determined by Student's *t*-test and Mann-Whitney test as appropriate

Results are expressed as number of patients (%), mean ± standard deviation or median (25th–75th percentile)

^a^Defined as the total number of defecations occurring during the treatment period divided by the total days of observation

^b^For patients who did not defecate during the treatment period, the number of hours of observation was considered

^c^Defined as the absence of defecation for at least 4 consecutive days

There were no differences between the groups with regard to the percent target caloric intake, gastric residue volume or prokinetic use (Additional file 1: Table S1). Additionally, there were no differences between the groups in the use of sedatives, opioids, neuromuscular blocking agents, vasoactive drugs or insulin (Additional file 1: Table S2). The nurse workload, assessed by the TISS-28 score, was not different between the groups (Table 3).

**Table 3:** Clinical outcomes of patients in both study groups

Variables	Intervention	Control	Mean difference	*p* value^a^
	(n = 44)	(n = 44)	(control – intervention) or relative risk (95 % CI)^a^	
*Primary outcome*				
ΔSOFA^b^	–4.0 (–6.0 to 0)	–1.0 (–4.0 to 1.0)	–1.907 (–3.683 to 0.13)^c^	0.036
SOFA at outcome assessment	4.0 (3.0 to 7.0)	5.5 (3.0 to8.7)	-	-
*Secondary outcomes*				
TISS-28 score				
Daily average	31.2 ± 6.41	30.7 ± 5.28	0.077 (–2.335 to 2.49)^c^	0.949
Total sum	419.0 (252.5 to 640.5)	433.0 (224.5 to 610.0)	24.4 (–73.8 to 122.7)^c^	0.622
Length of ICU stay (days)	16.0 (10.0 to 23.0)	16.0 (10.0 to 21.0)	–1.843 (–5.119 to 1.433)^c^	0.277
Length of hospital stay (days)	32.5 (18.2 to 49.5)	27.0 (17.0 to 56.7)	–3.19 (–28.275 to 21.894)^c^	0.804
New infection	19 (43.2)	12 (27.3)	1.673 (0.936 to 3.149)^d^	0.087
Number of new infections	0 (0 to 1.0)	0 (0 to 1.0)	1.627 (0.821 to 3.325)^d^	0.172
Pneumonia	17 (38.6)	10 (22.7)	1.79 (0.936 to 3.655)^d^	0.085
UTI	4 (9.1)	2 (4.5)	2.531 (0.508 to 17.677)^d^	0.264
CRBSI	2 (4.5)	2 (4.5)	0.815 (0.094 to 6.994)^d^	0.836
Bacteremia	5 (11.6)	9 (20.9)	0.397 (0.128 to 1.124)^d^	0.067
Severe sepsis/septic shock	12 (27.6)	14 (31.8)	0.842 (0.428 to 1.625)^d^	0.607
New organ dysfunction	23 (52.3)	24 (54.5)	0.932 (0.621 to 1.396)^d^	0.726
Ventilator-free days in 28 days	16.5 (11.0 to 21.7)	20.0 (13.2 to 24.0)	–1.371 (–4.569 to 1.826)^c^	0.396
Mortality				
Day 28	10 (22.7)	16 (36.4)	1.29 (0.973; 1.715)^d^	0.081
ICU	11 (25.0)	17 (38.6)	1.311 (0.975; 1.767)^d^	0.077
Hospital	13 (29.5)	19 (43.2)	1.294 (0.932; 1.803)^d^	0.129

Results are expressed as number (%), mean ± standard deviation or median (25th to 75th percentile)

^a^Generalized linear model with gaussian family with identity link or quasipoisson with log link, or binomial with log link as appropriate; models were controlled by APACHE score and SOFA at baseline

^b^ΔSOFA is the change in SOFA score between the date of enrollment (day 0) and the day of outcome assessment. Outcome was assessed at ICU discharge, death or day 15 after enrollment, whichever came first

^c^mean difference between groups estimated with delta method from a quasipoisson regression considering median APACHE and SOFA scores at baseline

^d^Relative risk

*APACHE* Acute Physiologic Chronic Health Evaluation, *CI* confidence interval, *CRBSI* catheter-related bloodstream infection, *ICU* intensive care unit, *SOFA* Sequential Organ Failure Assessment score, *TISS-28* Therapeutic Intervention Scoring System-28, *UTI* urinary tract infection

**Fig. 2:**
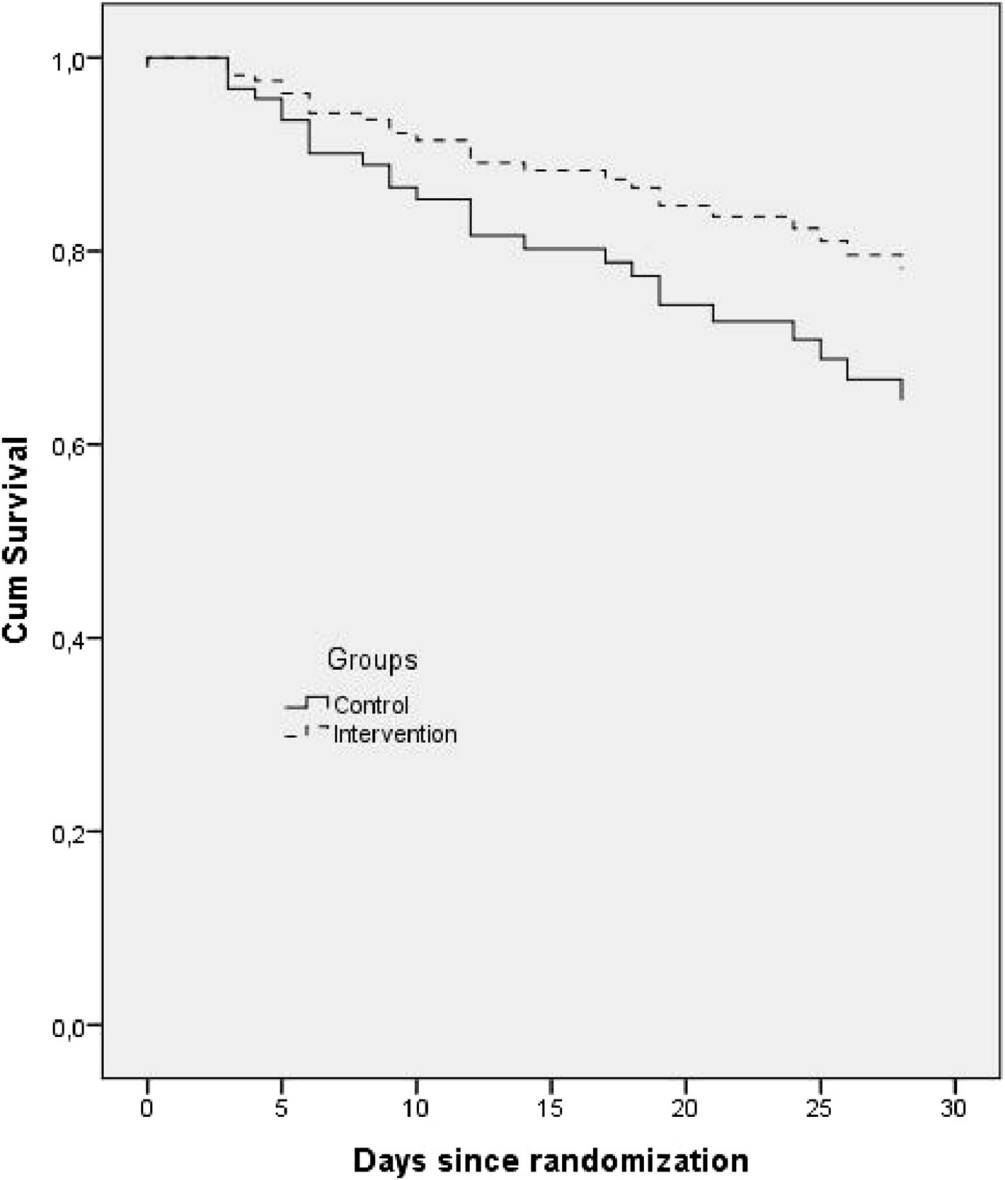
Kaplan-Meier curve demonstrating the 28-day survival of the ICU patients according to their group allocation. Cox proportional hazards analysis results following adjustment for the category of admission, APACHE score, age and SOFA score (hazard ratio 1.772, 95 % confidence interval 0.789 to 3.978.51; *p* = 0.166) are shown. *Dotted line* intervention group; *filled line* control group

The data pertaining to the primary and secondary outcomes are presented in Table 3. The median days for outcome assessment were 13.5 (7.2–19.7) days in the control group and 14.0 (8.0–20.7) days in the intervention group (*p* = 0.567). In the control group, 24 patients were assessed before day 14; 10 of them died during ICU stay. In the intervention group, 23 patients were assessed before day 14 and six of them died in the ICU, with no differences between the groups (*p* = 0.260). For the intention-to-treat analysis, the reduction in SOFA scores was greater in the intervention group (–4.0 (–6.0 to 0) versus –1.0 (–4.0 to 1.0), *p* = 0.036). The differences between the individual components of SOFA scores are presented in Additional file 1: Table S3. There were no differences in any of the secondary outcomes. There was no significant improve in survival in the intervention group (*p* = 0.166; Fig. 2). Five patients remained on MV for less than 3 days, including three in the intervention group and two in the control group. The per-protocol analysis confirmed that the difference in ΔSOFA was significant (Additional file 1: Tables S4 and S5).

AEs were more frequent among the patients in the study group, with higher incidences of diarrhea, abdominal distension and vomiting. There were few serious adverse events reported in either group, and their incidences did not significantly differ between the groups (Table 4).

**Table 4:** Adverse events of the patients in both study groups

Variable	Intervention	Control	*p* value^a^
	(n = 44)	(n = 44)	
Adverse events			
Patients with AE	43 (97.7)	41 (93.2)	0.306
AE per patient	4.5 (3.0–8.0)	3.0 (1.0–5.7)	0.016
AE per patient/day	0.35 (0.2–0.5)	0.2 (0.1–0.3)	0.002
Diarrhea^b^	41 (93.2)	26 (59.1)	<0.001
Diarrhea, % days	17.0 (9.3–25.0)	6.3 (0–14.7)	<0.001
Diarrhea, days with	2.0 (1.0–4.0)	1.0 (0–2.0)	<0.001
Gastric reflux ≥500 ml/day^b^	18 (40.9)	19 (43.2)	0.829
Gastric reflux, ml/day	76.5 (29.0–117.7)	61.5 (22.2–112.2)	0.350
Abdominal distension^b^	8 (18.2)	2 (4.5)	0.044
Vomiting^b^	11 (25.0)	4 (9.1)	0.047
Hypernatremia^b^	4 (9.1)	7 (15.9)	0.334
Hypokalemia^b^	24 (54.5)	18 (40.9)	0.200
Hypomagnesemia^b^	13 (29.5)	18 (40.9)	0.265
Decubitus ulcer^b^	4 (9.1)	5 (11.4)	0.725
Dermatitis^b^	2 (4.5)	1 (2.3)	0.557
Serious adverse event	5 (11.4)	3 (6.8)	0.461
Ogilvie syndrome, number of patients	2 (4.5)	0 (0)	0.153
Gastrointestinal bleeding, number of patients	1 (2.3)	1 (2.3)	_~_1.00
Severe hypomagnesemia	1 (2.3)	0 (0)	>0.99
Severe hypokalemia	1 (2.3)	1 (2.3)	_~_1.00
Severe hyponatremia	0 (0)	1 (2.3)	>0.99

Results are expressed as number (%) or median (25th–75th percentile)

^a^Chi-square and Mann-Whitney test as appropriate

^b^Results reported as number of patients with event

*AE* adverse event

## Discussion

In this phase II study, we demonstrated that the use of laxative therapy with lactulose was effective in inducing defecation in critically ill, mechanically ventilated patients. Our results suggest that laxative therapy is a safe procedure. The protocol was not accompanied by an increase in the incidence of serious AEs, although a slight increase in minor AEs, such as diarrhea, abdominal distension and vomiting, occurred in the intervention group. In addition, the nursing staff workload was not affected by the treatment. Laxative therapy aimed at daily defecation was associated with a greater reduction in the SOFA score at discharge, death or after 14 days of treatment.

Some studies have related constipation to the failure to wean from MV, to an increased length of hospitalization, and even to the degree of organ dysfunction and death [1, 9, 11, 13]. van der Spoel et al. published a randomized clinical trial assessing the impacts of laxatives, lactulose and polyethyleneglycol on early defecation [13]. These authors demonstrated a shorter ICU stay for the group receiving lactulose, and a multivariate analysis identified APACHE II and the number of defecations as independent predictors of mortality. This study, however, did not evaluate the impacts of laxative use on organ dysfunction or on the development of new infections. Masri et al. randomized 100 patients to receive or to not receive lactulose only during the first 3 days of ICU admission as a prophylaxis for constipation [21]. The times of the first bowel movements differed between the groups. They were not able to show any significant differences in the other outcomes except for the shorter duration of MV observed in those patients who defecated after the fifth day. However, they did not report the MV results on the ventilator-free days. Neither study was designed to assess the impacts of daily laxation treatment. Our study focused on daily laxation, and our results suggest that the intervention might be associated with a greater reduction in organ dysfunction.

The association of obstipation with poor prognosis has been suggested previously [1, 2, 9, 11, 12]; however, a causality effect has never been demonstrated. Our study aimed to assess the efficacy of our protocol and its impact on the degree of organ dysfunction and not the underlying physiological mechanisms. Thus, we cannot explain our findings but rather generate hypotheses. First, the use of laxation may have reduced intestinal flora overgrowth, which may have contributed to reduced bacterial translocation and expression of bacterial products in the mucosal epithelium, decreasing the production of proinflammatory mediators as previously suggested [22–24]. Obstipation may also have contributed to abdominal hypertension. Abdominal compartment syndrome has been associated with worse prognosis in critically ill patients [25–27]. Possible mechanisms are a worsening in respiratory function [28] and the interference with the intra-abdominal or the diaphragmatic work of breathing [29] as well as reduced splacnic perfusion [30].

As expected, there was an increased incidence of diarrhea in the laxative therapy group as well as a slightly higher incidence of abdominal distention and vomiting. There was no increase in the objective measurement of gastric reflux. Two of the patients in the intervention group had Ogilvie’s syndrome. Although lactulose is associated with abdominal distention, its use has not been associated with a higher incidence of Ogilvie’s syndrome in a previous study [13]. This syndrome has been associated with many conditions that are prevalent in critically ill patients [31]. Moreover, lactulose has been widely used in ICUs as part of the treatment of hepatic encephalopathy with similar doses and Ogilvie’s syndrome is not reported as a complication of this treatment.

Our study has some strengths. This was a prospective, randomized study including consecutive patients. Our protocol was carefully designed to produce daily evacuation, and the ICU team was informed of the protocol steps, resulting in well-separated study groups. We conducted a comprehensive evaluation of the concomitant medications to assure that the randomized groups were balanced. We also assessed the safety of the intervention, including the occurrence of AEs and the impact of this strategy on the nurse workload in the ICU.

This study also has some limitations. It is a single-center, phase II trial with a small sample size, including only mechanically ventilated patients receiving enteral nutrition, which limits the external validation of the results and the evaluation of mortality or of other long-term outcomes. The nonblinded design also may have caused bias. A blinded study, using placebo, would not be feasible. Lactulose is difficult to blind due to its natural effects. Moreover, the intervention was daily defecation rather than to test the effects of lactulose. Thus, the use of enemas in the absence of bowel movement only in the intervention group would have been impossible if the study was nonblinded. The use of a placebo enema in the control group could have generated bowel stimulus and consequent evacuation. However, this bias was minimized because the study staff were not directly responsible for the clinical management of the patients. We also did not evaluate the presence of intra-abdominal hypertension. Possible differences between the groups in this parameter would have helped to better understand one of the possible physiological mechanisms by which treating obstipation would reduce organ dysfunction. We could also have used ultrasonography to evaluate the degree of fecal impaction and to assess bowel movements, as well as to objectively evaluate abdominal distension, one of our major potential AEs associated with lactulose use. We recognize that abdominal distension was only subjectively assessed and this might be biased because of the nonblinded nature of our study. However, abdominal distension was more commonly described in the intervention group, which is an indirect indicator of reduced risk of bias. Another limitation is the absence of data on cumulative fluid balance. Capillary leak and excessive resuscitation with crystalloids are associated with increased gut wet-to-dry ratio and bowel edema resulting in decreased bowel contractility and abdominal hypertension [32]. If fluid balance was different between the groups we could hypothesize that there was a higher chance of constipation as well as abdominal compartment syndrome and this would impact in the effectiveness of our protocol. However, fluid balance is highly related to severity of illness and the severity of illness, even if not significant, seems to be greater in the intervention group.

Thus, our results should be interpreted with caution. Future studies with adequate sample sizes are needed to further understand the effects of this therapy on mortality in critically ill patients.

## Conclusion

Our laxative therapy protocol was able to improve daily defecation in mechanically ventilated patients and was associated with a greater reduction in the SOFA score at discharge, death or after 14 days despite a small increase in nonserious AEs.

## Key messages

- Obstipation is a common problem in critically ill patients as demonstrated by the time for first defecation and number of defecations per day in our control group.
- Treatment with lactulose was effective in promoting daily defecation. Patients in the treatment arm had a higher number of defecations per day, a shorter period for the first defecation and a lower percentage of days without defecation throughout the study.
- Although there was no difference in the survival time or in mortality, the daily defecation protocol was associated with a greater reduction in SOFA scores as compared with the control arm.
- The intervention was also associated with an increase in nonserious AEs, such as diarrhea, abdominal distension and vomiting.

## Additional file

Additional file 1. **Supplementary material.** (DOCX 45 kb)

## Abbreviations

AE: Adverse event
APACHE: Acute Physiologic Chronic Health Evaluation
ICU: Intensive care unit
MV: Mechanical ventilation
SOFA: Sequential Organ Failure Assessment
TISS: Therapeutic Intervention System Score
