# Supplementary material for: Daily laxative therapy reduces organ dysfunction in mechanically ventilated patients: a phase II randomized controlled trial
Source: Crit Care. 2015 Sep 16;19(1):329. doi: 10.1186/s13054-015-1047-x (PMC4572636; doi:10.1186/s13054-015-1047-x)
Supplement: Supplementary file 1 — Supplementary material. (DOCX 45 kb) [file 13054_2015_1047_MOESM1_ESM.docx]

**Supplemental materials**

**Daily laxative therapy reduces organ dysfunction in mechanically ventilated patients: a phase II randomized controlled trial.**

Rodrigo P. de Azevedo, MD; Flávio G.R. Freitas, MD, PhD; Elaine M. Ferreira, RN; Luciano C.P. Azevedo, MD, PhD; Flávia R. Machado, MD, PhD

**Laxative protocol description**

Intervention group

The intervention group received lactulose at initial doses of 20 mL every 8 h. The goal was to produce 1-2 bowel movements per day. During the first day, if the patient did not evacuate within 48 h prior to inclusion in the study or if this information was not available, a rectal enema was administered (glycerin enema, 20%, 10 mL/Kg or phosphonate enema, 115 mL) up to 3 times per day and discontinued only when the patient produced stool.

During the first 2 days of the study, the lactulose was administered at a fixed dose. On the third day and thereafter, if the patient remained without defecation, up to three daily rectal enemas per day were prescribed, and the dose of lactulose was increased by 50% per day up to a dose of 45 mL every 8 h. At any time, the assistant physicians were permitted to attempt to mechanically remove the feces. If the constipation persisted despite the maximum doses of lactulose and enema, the presence of other conditions, such as Ogilvie's syndrome, was investigated, and specific treatments were initiated at the discretion of the attending physician.

At any time, if the patient had more than 2 bowel movements per day, the lactulose was discontinued for 24 h and re-initiated at 50% of the initial dose, with a minimum dose of 5 mL every 8 h. Patients in the intervention group who remained with diarrhea after the lactulose was withdrawn were treated according to the local protocols for diarrhea. For the patients with abdominal pain or with distention, the lactulose was withheld and resumed after improvement of the symptoms. If the lactulose intolerance persisted, this medication was discontinued, and attempts to reintroduce it were carried out ​​every two days. During this period, the patients who had not defecated for over 24 h received a rectal enema. As previously mentioned, this procedure could be repeated up to 3 times daily. If enteral nutrition was discontinued for any reason, the lactulose was withheld until the re-initiation of the enteral nutrition with an effective delivery of above 20% of the basal energy expenditure.

Control group

The management of constipation was based on local protocols. Patients who had not defecated for 5 days after of the onset of enteral nutrition received a daily rectal enema (glycerin enema, 20%, 10 mL/Kg or phosphonate enema, 115 mL) after the mechanical removal of any rectal fecal impaction, if present. If the patient remained without defecation, the presence of other conditions, such as Ogilvie's syndrome, was investigated, and specific treatments were performed at the discretion of the attending physician. In such cases, the patient was administered lactulose. The approach for treating diarrhea in the patients in the control group was based on a local protocol.

The ICU multidisciplinary team, particularly the nursing staff, was informed of the main steps of the protocol. Posters were printed and widely distributed. The protocol team was responsible for prescribing the adequate dose of lactulose or enemas to both group, if indicated.

**Nutritional support**

In both groups, nutritional support was based on local protocols. A nutritionist, together with the assistant intensivist, plans the dietary daily target during the multidisciplinary rounds. The overall calorie target is calculated based on 20-25 kcal / kg in the acute phase of injury and 25-30 kcal / kg in the recovery phase. Enteral feeding is started preferably in the first 48 hours after ICU admission if the patient is hemodynamically stable with adequate perfusion parameters. Our protocol includes an open diet system and intermittent feeding, with the total caloric target divided in 6 or 8 feedings. Diet volume is increased according to tolerance, beginning with 600 kcal on Day 1, 900 kcal on Day 2, 1200 kcal on Day 3 and the calculated target on Day 4. Diet on the 1^st^ day has a caloric density of 1Kcal / ml with no fiber in its composition. If tolerated, in the subsequent days the diet is changed to a calorie density of 1,5Kcal / ml with fibers.

Gastric reflux is routinely checked before each diet. If volume is less than 200 ml, the diet is infused as prescribed. If the volume is greater than 200 the diet is withheld until the next schedule time. If the volume is over 500 ml, diet is withheld and the enteral feeding tube is left open for drainage. After the first day without receiving at least 50% of the prescribed diet because of gastric reflux, metoclopramide is started. In refractory cases, a combined therapy with bromopride is initiated.

**Data collection**

For all patients, we recorded general descriptive data, such as the demographic data, admission diagnosis, comorbidities, Acute Physiological Assessment and Chronic Health (APACHE II) score, [^1^](#_ENREF_1) and Sequential Organ Dysfunction Assessment (SOFA) score at the time of study inclusion and on subsequent days. [^2^](#_ENREF_2) The time between hospital admission and ICU admission and the time between ICU admission and enrollment were also recorded. Nutrition support data were collected during the protocol period and included the percentage of target calories actually received, the gastric residue volume, the number of days without enteral nutrition and the use of prokinetics. Other variables recorded included the uses of sedatives and analgesics, neuromuscular blockers, vasoactive drugs and insulin.

**Definitions**

We defined severe sepsis and septic shock according to the American College of Chest Physicians and the Society of Critical Care Medicine Consensus Conference criteria.[^3^](#_ENREF_3) All infections were defined according to the Center for Disease Control and Prevention.[^4^](#_ENREF_4) Organ dysfunction definitions were used for both severe sepsis and to assess new dysfunctions due to any other causes. Organ dysfunctions were not secondary to chronic disease and included one of the following: cardiovascular – hypotension with systolic blood pressure below 90 mmHg or mean arterial blood pressure below 65 mmHg; respiratory – arterial oxygen partial pressure/oxygen inspiratory fraction (PaO_2_/FiO_2_) ratio < 250; renal – creatinine > 2.0 mg/dL, an increase of greater than 50% in the baseline levels or a need for renal replacement therapy; neurological – a change in mental status (Glasgow Coma Scale < 13); hematological – platelet count < 100,000 cells/mm^3^; hepatic – plasma bilirubin > 2.0 mg/dL.

**Adverse events definition**

We defined adverse events (AEs) as the following: diarrhea – 3 or more episodes of bowel movements per day; elevated gastric residuals – gastric residuals ≥ 500 mL in 24 h; vomiting – reflux of nutrients or of gastric fluids into the mouth noted on the patient’s chart by the nursing staff; abdominal distention – abdominal enlargement with the increased tension of the abdomen verified daily by the study team or if any of the assistant physicians register the event in the patients chart; hypernatremia – serum sodium > 145 mmol/L; hypokalemia – serum potassium < 3.5 mmol/L; hypomagnesemia – serum magnesium < 1.8 mmol/L; pressure ulcer – the presence of a pressure ulcer on the patient’s backside region; and dermatitis – the presence of dermatitis in the inguinal region. We recorded the total number of patients with AEs for each group. Diarrhea was also reported as the percentage of days with the condition. We recorded the total number of severe adverse events (SAEs) for each group.

**Table S1. Nutritional data**

| **Variable** | **Intervention**  **(n = 44)** | **Control**  **(n = 44)** | **p value*** |
| --- | --- | --- | --- |
| Calories administered, % target | 84.0 (52.0-96.0) | 77.5 (64.5-87.5) | 0.447 |
| Days without enteral feeding | 5.0 (0-12.0) | 0 (0-11.0) | 0.360 |
| Prokinetics, patient use | 32 (72.7) | 31 (70.5) | 0.813 |
| Prokinetics, days of use | 50.0 (32.5-78.75) | 38.0 (12.0-67.0) | 0.099 |

Results are expressed as number (%) or median (p25-p75). *Chi-square and Mann-Whitney test as appropriate.

**Table S2. Adjunctive treatment characteristics**

| **Variable** | **Intervention**  **(n = 44)** | **Control**  **(n = 44)** | **p value**** |
| --- | --- | --- | --- |
| Midazolam, patient use | 29 (65.9) | 36 (81.8) | 0.089 |
| Midazolam, days of use | 29.0 (15.5-49.0) | 23.0 (11.25-51.7) | 0.428 |
| Midazolam, mg/day* | 44.0 (8.5-81.0) | 17.0 (2.0-88.7) | 0.245 |
| Propofol, patient use | 29 (65.9) | 24 (54.5) | 0.276 |
| Propofol, days of use | 23.5 (12.7-50.0) | 21.0 (11.0-54.5) | 0.879 |
| Propofol, mL/day* | 18.0 (7.5-105.5) | 41.5 (6.5-95.7) | 0.768 |
| Thiopental, patient use | 5 (11.4) | 8 (18.2) | 0.367 |
| Thiopental, days of use | 28.4±20.9 | 34.00±26.1 | 0.695 |
| Thiopental, g/day* | 1.0 (0.5-2.0) | 1.5 (0.5-2.5) | 0.537 |
| Fentanyl, patient use | 42 (95.5) | 40 (90.9) | 0.398 |
| Fentanyl, days of use | 41.8±28.9 | 51.3±26.5 | 0.131 |
| Fentanyl, mg/day* | 1059.5 (151.5-2754.0) | 1583.0 (183.5-216.0) | 0.466 |
| Tramadol, patient use | 38 (86.4) | 32 (72.7) | 0.113 |
| Tramadol, days of use | 45.0 (20.0-71.5) | 35.5 (20.0-51.5) | 0.454 |
| Tramadol, mg/day* | 106.5 (51.0-151.25) | 77.0 (44.0-161.2) | 0.190 |
| Morphine, patient use | 7 (15.9) | 6 (13.6) | 0.764 |
| Morphine, days of use | 28.0 (10.0-80.0) | 23.0 (16.5-32.0) | 0.731 |
| Morphine, mg/day* | 16.0 (7.0-94.0) | 7.5 (4.25-34.25) | 0.366 |
| NMB, patient use | 19 (43.2) | 14 (31.8) | 0.271 |
| NMB, days of use | 15.0 (7.0-38.0) | 11.0 (4.0-31.5) | 0.397 |
| Norepinephrine, patient use | 30 (68.2) | 32 (72.7) | 0.640 |
| Norepinephrine, days of use | 30.5 (20.7-62.5) | 36.0 (18.5-74.0) | 0.578 |
| Dobutamine, patient use | 9 (20.5) | 15 (34.1) | 0.151 |
| Dobutamine, days of use | 49.4±36.7 | 48.2±30.8 | 0.930 |
| Epinephrine, patient use | 4 (9.1) | 3 (6.8) | 0.694 |
| Epinephrine, days of use | 20.2±14.0 | 39.3±32.0 | 0.320 |
| Insulin, patient use | 34 (77.3) | 32 (72.7) | 0.622 |
| Insulin, days of use | 37.0 (22.2-81.2) | 45.0 (18.0-83.7) | 0.630 |
| Insulin, UI/day* | 7.5 (2.0-24.2) | 6.0 (2.0-21.2) | 0.685 |

NMB – neuromuscular blockers. *Only patients who received the drug. Results are expressed as number (%) or median (p25-p75). **Chi-square and Mann-Whitney test as appropriate.

**Table S3. Individual deltaSOFA scores, according to study group**

| **Individual deltaSOFA scores** | **Intervention**  **(n = 44)** | **Control**  **(n = 44)** | **p value** |
| --- | --- | --- | --- |
| Respiratory | 0(-1.0 to 0) | 0(-1.0 a 1.0) | 0.454 |
| Hematologic | 0(-1.0 to 0) | 0(-1.0 to 0) | 0.176 |
| Hepatic | 0(0 to 0) | 0(0 to 0) | 0.789 |
| Cardiovascular | 0(-3.5 to 0) | 0(-3.0 to 0) | 0.300 |
| Neurological | 0(-1.0 to 0) | 0(0 to 1.0) | 0.110 |
| Renal | 0(0 to 0) | 0(0 to 2) | 0.263 |

Per-protocol analysis – main results

**Table S4. Effectiveness of the protocol – per-protocol analysis**

| **Variable** | **Intervention**  **(n = 41)** | **Control**  **(n = 42)** | **p-value*** |
| --- | --- | --- | --- |
| **Defecation ratio*** | 1.3±0.42 | 0.7±0.60 | <0.0001 |
| **Time to first defecation- hours** | 14.0 (4.0-24.0) | 96.0 (47.5-126.5) | <0.0001 |
| **Days without defecation- % days** | 33.8±15.1 | 61.7±24.3 | <0.0001 |
| **Enema administration- % days** | 22.2 (12.5-28.3) | 5.4 (0-11.9) | <0.0001 |

*Defecation ratio = total number of defecations during observation period/total days of observation. Results are expressed as mean ± standard deviation or median (p25-p75). *Student's t-test and Mann-Whitney test as appropriate.

**Table S5. Clinical outcomes - per-protocol analysis**

| **Variables** | **Intervention**  **(n = 41)** | **Control**  **(n = 42)** | **p value*** |
| --- | --- | --- | --- |
| **Primary outcome** |  |  |  |
| SOFA D14** | 4.0 (3.0-7.0) | 6.0 (3.0-9.5) | 0.101 |
| ΔSOFA D14 | -4.0 (-6.0-0.5) | -1.0 (-4.0-1.25) | 0.030 |
| **Secondary outcomes** |  |  |  |
| SOFA D7** | 5.0 (4.0-7.5) | 5.5 (4.0-8.75) | 0.789 |
| ΔSOFA D7 | -1.0 (-5.0-1.0) | -1.0 (-3.25-1.0) | 0.402 |
| TISS-28 score |  |  |  |
| Daily average | 30.2 ± 5.42 | 31.0 ± 5.13 | 0.644 |
| Total sum | 437.0 (305.5-646.0) | 457.5 (241.7-613.5) | 0.455 |
| Length of ICU stay (days) | 17.0 (12.5-23.5) | 16.5 (10.0-21.5) | 0.384 |
| Length of hospital stay (days) | 32.0 (18.5-49.0) | 28.0 (17.0-58.2) | 0.927 |
| Patients with new infection | 19 (46.3) | 12 (27.3) | 0.094 |
| Number of new infections | 0 (0-1.0) | 0 (0-1.0) | 0-149 |
| Pneumonia*** | 17 (41.5) | 10 (23.8) | 0.086 |
| Urinary tract infection*** | 4 (9.8) | 2 (4.8) | 0.380 |
| CRBSI*** | 2 (4.9) | 2 (4.8) | 0.980 |
| Bacteremia*** | 5 (12.5) | 9 (20.0) | 0.261 |
| Severe sepsis/septic shock*** | 12 (29.7) | 14 (33.3) | 0.690 |
| New organ dysfunction*** | 23 (56.1) | 24 (57.1) | 0.923 |
| Ventilator-free days | 16.5 (11.0-21.7) | 20.0 (13.2-24.0) | 0.271 |
| Mortality |  |  |  |
| Day 28 | 9 (22.0) | 16 (38.1) | 0.109 |
| ICU | 10 (24.4) | 17 (40.5) | 0.118 |
| Hospital | 11 (26.8) | 19 (45.2) | 0.081 |

SOFA - Sequential Organ Failure Assessment score; CRBSI – catheter-related bloodstream infection; ICU – intensive care unit. Results are expressed as number (%), mean ± standard deviation or median (p25- p75). * Chi-square test, Student's t-test and Mann-Whitney test as appropriate. **If patient died or was discharged prior to D7 or D14, SOFA score at discharge or death was used. ***Results reported as number of patients with event.

References

1 Knaus WA, Draper EA, Wagner DP, et al. APACHE II: a severity of disease classification system. Crit Care Med 1985; 13:818-829

2 Vincent JL, Moreno R, Takala J, et al. The SOFA (Sepsis-related Organ Failure Assessment) score to describe organ dysfunction/failure. On behalf of the Working Group on Sepsis-Related Problems of the European Society of Intensive Care Medicine. Intensive Care Med 1996; 22:707-710

3 American College of Chest Physicians/Society of Critical Care Medicine Consensus Conference: definitions for sepsis and organ failure and guidelines for the use of innovative therapies in sepsis. Crit Care Med 1992; 20:864-874

4 Horan TC, Andrus M, Dudeck MA. CDC/NHSN surveillance definition of health care-associated infection and criteria for specific types of infections in the acute care setting. Am J Infect Control 2008; 36:309-332
